# Development of a New Tomato Sauce Enriched with Bioactive Compounds Through the Use of Processing By-Products and Vegetables

**DOI:** 10.3390/foods14122037

**Published:** 2025-06-09

**Authors:** Enrico Maria Milito, Lucia De Luca, Giulia Basile, Martina Calabrese, Antonello Santini, Sabato Ambrosio, Raffaele Romano

**Affiliations:** 1 Department of Agricultural Sciences, University of Naples Federico II, Piazza Carlo di Borbone I, 80055 Portici, NA, Italy; enricomaria.milito@unina.it (E.M.M.); lucia.deluca@unina.it (L.D.L.); giulia.basile@unina.it (G.B.); martina.calabrese@unina.it (M.C.); sabato.ambrosio@yahoo.it (S.A.); rafroman@unina.it (R.R.); 2 Department of Pharmacy, University of Napoli Federico II, Via Domenico Montesano 49, 80131 Napoli, NA, Italy

**Keywords:** food waste, tomatoes, food processing, new products development, nutritional enrichment

## Abstract

In recent years, the development of nutritionally enhanced foods with reduced environmental impact has gained significant importance. This study aimed to produce four types of tomato sauces: traditional, whole (including peels and seeds), traditional with added vegetables, and whole with added vegetables. The vegetables included in the latter two variations were pumpkin, carrot, basil, and oregano. The sauces were analyzed for various parameters, such as soluble solids content, viscosity, pH, reducing sugars, titratable acidity, color, sodium, calcium, potassium, magnesium content, total polyphenols, lycopene, beta-carotene, antioxidant activity, dietary fiber content, vitamin C, and volatile organic compounds. Results showed that whole tomato sauces had up to 80% more polyphenols (270.40 vs. 150.30 mg GAE/kg f.w.) and 30% higher DPPH antioxidant activity (87.07 vs. 66.96 µmol TE/100 g) compared to traditional sauces. Vegetable enrichment, particularly with pumpkin and carrot, significantly increased β-carotene levels (up to 68.67 mg/kg f.w.). Incorporating peels and seeds boosted the bioactive components, and adding vegetables provided an additional nutritional benefit. These findings highlight how waste recovery can contribute to the development of products with enhanced health benefits, offering a sustainable approach to food production.

## 1. Introduction

The global population is increasing rapidly and is projected to reach 9.6 billion by 2050, up from 6.7 billion today. This growth will significantly increase the global demand for food [1]. However, feeding a growing population remains a major challenge. The 2030 Agenda for Sustainable Development, adopted by governments, businesses, and civil society in partnership with the United Nations, emphasizes the need to “do more and better with less” by promoting sustainable consumption and production. The Food and Agriculture Organization (FAO) estimates that global agricultural production must increase by 70% to meet future demand. In this context, tomatoes emerge as a key crop. Tomatoes are among the most widely consumed vegetable crops in the world, thanks to their ease of processing and preservation, which allows for year-round availability. Italy is the leading producer in Europe, producing over 5 million tons, followed by Spain with 2.95 million tons, and Portugal with 1.56 million tons [2]. Nationally, tomatoes are essential to Italian cuisine and renowned for their sensory and nutritional qualities. They are available both fresh and in processed forms, including peeled tomatoes, pulp, puree, concentrate, and sauces [2]. However, tomato processing produces significant by-products, accounting for approximately 2–5% of the total processed mass [3]. These by-products, primarily peels and seeds, are often referred to as tomato waste [4,5]. Traditionally, they have been used as animal feed, particularly in cattle silage and pig diets [6]. In recent years, there has been growing interest in exploring alternative uses for these materials due to their high content of bioactive compounds [3]. Tomato by-products, in particular, are known to be rich in polyphenols, lycopene, β-carotene, vitamin C, and dietary fiber [7,8]. Despite extensive research, the full potential of these compounds has yet to be fully realized. Dietary fibers are well known for their health benefits, including reducing the risk of obesity, type II diabetes, cancer, and digestive disorders [8]. Polyphenols, on the other hand, are valued for their antioxidant properties, which help combat oxidative stress and chronic diseases. Moreover, recent studies suggest that polyphenols may also improve insulin resistance and support weight management due to their hypoglycemic and anti-inflammatory effects [9]. Among carotenoids, lycopene and β-carotene are the most extensively studied. Lycopene contributes to preventing cardiovascular diseases and cancer, supporting metabolism, minimizing intestinal damage, and reducing immunoglobulin A leakage [10]. β-Carotene (provitamin A) plays a role in lipid and cholesterol metabolism and has anti-inflammatory and anticancer properties [11]. Given the rising prevalence of cardiovascular diseases, cancer, and type II diabetes, the food industry is increasingly focused on developing healthier products that align with consumer preferences. According to Moors [12], functional foods not only provide essential nutrients but also support specific bodily functions, helping prevent diseases and addressing public health challenges. However, the effectiveness of functional foods is highly dependent on the bioavailability of bioactive compounds. In this process, the food matrix plays a key role: the choice of an appropriate carrier and the use of technologies capable of preserving the bioactive form until consumption are key elements in maximizing the benefits [13]. Among the promising technologies, homogenization has proven effective in improving bioavailability by enhancing the release and absorption of these compounds [14,15]. These aspects will be explored in the following sections to highlight the opportunities offered by such processes in the nutritional enhancement of food. This study introduces an innovative approach using advanced continuous ultra-shredding technology designed to optimize the structure of tomato sauce and enhance the release of bioactive compounds. The objective of this study was to develop and compare four types of tomato sauces: a traditional sauce, a traditional sauce enriched with vegetables, a whole-tomato sauce (including skins and seeds), and a whole-tomato sauce enriched with vegetables. This innovative approach involved the use of ultra-shredding to recover and incorporate tomato skins and seeds, by-products typically discarded during processing, hereby enhancing the nutritional profile of the final product. The analysis focused on evaluating the chemical and physical properties of each variant, with particular attention to the effects of whole-tomato processing and the nutritional impact of vegetable enrichment.

## 2. Materials and Methods

### 2.1. Chemicals

All solvents and reagents used for the experiments were purchased from Sigma–Aldrich Co., (Milan, Italy).

### 2.2. Materials

Tomato *cultivar* SV880, pumpkin (*cultivar Mantovana*), basil (*cultivar Genovese*), oregano (*cultivar Ricigliano*), orange carrot (*cultivar Carota Novella di Ispica*), and white onion *(cultivar Bianca di Pompei*) have been used for sample preparation of the tomato sauce. The vegetables used were purchased from the local market.

### 2.3. Tomato Sauce Preparation

**Figure 1:**
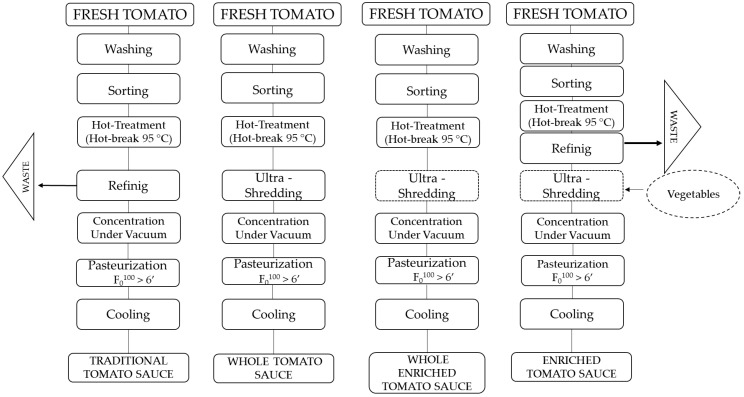
Production process of the traditional tomato sauce (TS), traditional enriched tomato sauce (ETS), whole tomato sauce (WTS), and whole enriched tomato sauce (WETS).

Tomato sauce samples were produced at the company La Torrente S.r.l. (Sant’Antonio Abate, Naples, Italy). After washing and manual and optical selection, the tomatoes were crushed and subjected to a hot-break treatment at 85 °C. For the traditional tomato sauce (TS), a refining step was implemented to remove the skins and seeds, while for the whole tomato sauce (WTS), this refining step was bypassed. For the vegetable-enriched variants, pumpkin, carrot, oregano, onion, and basil were added to the traditional or whole tomato sauce to obtain the traditional enriched tomato sauce (ETS) and the whole enriched tomato sauce (WETS). The recipe has been reported in Table 1 and the production process in Figure 1. The mixture underwent ultra-grinding using a Robot Comitrol^®^ 1700 (Urschel Laboratories, Chesterton, IN, USA), ensuring complete homogenization of the ingredients. The samples were then concentrated to standardize the content of total soluble solids to the legislative values for the definition of tomato sauce [16] and subsequently pasteurized at 95 °C for 40 min. The samples were analyzed after production.

### 2.4. pH, Total Soluble Solids, Dry Weight, and Viscosity

The determination of pH was carried out by a pH meter Crison Basic 20+ (Barcelona, Spain), according to the procedure described by the Official Methods [17]. The determination of the total soluble solids was carried out using a portable refractometer from SperScientific (Scottsdale, AZ, USA) according to the procedure described by the Official Methods [17]. The values are expressed in °Brix, which is defined as the concentration (%) of total soluble solids in solution when measured at 20 °C. The determination of the dry matter content was carried out by drying 3 g of sample at 100 °C until a constant weight. The results are expressed as weight/weight percentages of dry matter (% *w*/*w*).

The viscosity was measured in processed tomatoes according to the Bostwick method [18] using a Bostwick LS100 consistometer (Laboscientifica, Parma, Italy). The results were expressed as Bostwick degree (cm/30 s).

### 2.5. Titratable Acidity, Reducing Sugar and Fat Content

The titratable acidity and reducing sugar content were evaluated using the official method [17]. The titratable acidity was expressed as a percentage, indicating the amount of citric acid monohydrate to the total fresh weight of the sample, while the reducing sugars content was expressed as % of reducing sugars on fresh weight.

The lipids were extracted according to [19]. Briefly, the sample was subjected to three extractions with 16 mL of an acetone/hexane solution (40/60, *v*/*v*). The mixture was centrifuged at 3500 g for 5 min, and the supernatant phases (lipophilic fraction) were collected, dehydrated in a rotavapor, and the collected fat was stored at −20 °C until analysis. Fat yield was expressed as g fat/100 g sample.

### 2.6. Mineral Content

Mineral concentration was measured according to the method described by [20] with some modifications. About 1.0 g of the sample from each pass was weighed and placed in a muffle furnace at 550 ± 10 °C for 24 h. The ashes were dissolved in (10 mL) with a 1% nitric acid solution. Subsequently, the solution was filtered and subjected to spectroscopic analysis for the determination of mineral salts. Mg, Ca, K, and Na, expressed as parts per million (ppm), were quantified with a ContrAA 800 atomic absorption spectrometer (Analytik Jena AG, Jena, Germany), equipped with a D2 lamp background correction system and an air–acetylene flame. The measurements were carried out at wavelengths of 285.2 nm for Mg, 422.7 nm for Ca, 766.5 nm for K, and 589.6 nm for Na. The results are expressed as mg/100 g f.w.

### 2.7. Total Phenolic Compound and Antioxidant Activity Determination

#### 2.7.1. Extraction

Three grams of samples were added to 30 mL of 70% methanol, vortexed for 1 min and centrifuged at 3500 g using a PK 131 (ALC International S.r.l., Milano, Italy) for 10 min at 25 °C. The supernatant was stored at −20 °C until the analysis.

The same extracts were subsequently used for antioxidant activity assays.

#### 2.7.2. Spectrophotometric Assay

The total phenolic content in the hydrophilic fraction was determined using the Folin–Ciocalteu assay reported by Sacco et al. [21] and Rigano et al. [22]. The absorbance was read at 765 nm using a UV-1601PC UV–visible scanning spectrophotometer (Shimadzu, Milan, Italy), and the TPC was determined using a calibration curve of gallic acid in the linearity range of 10–50 μg mL^−1^ (R^2^ = 0.99). The results were expressed as mg gallic acid equivalents (GAEs)/100 g of sample fresh weight (mg/kg f.w.).

The antioxidant activity of the hydrophilic extracts was determined via ABTS●+ and DPPH assays. A 2,2′-azinobis-3-ethyl-benzothiazoline-6-sulfonic acid (ABTS●+) assay was performed as described by Luterotti et al. [23], with modifications. A quantity of 400 μL of diluted extract was mixed with 3 mL of the ABTS●+ working solution. The solution was kept in the dark for 5 min, and the absorbance was measured at 751 nm using a spectrophotometer. The antiradical activity was calculated using a Trolox calibration curve in the linearity range of 10–200 μM (R^2^ = 0.99). The results are expressed as μmol Trolox equivalent (TE)/100 g sample.

The 2,2-diphenyl-1-picrylhydrazyl (DPPH) assay was performed following the method proposed by Espinosa-Pardo et al. [24], with some modifications. An aliquot of 1 mL of diluted extract was mixed with 3 mL of DPPH reagent 0.1 mM. After 5 min of incubation at ambient temperature in the dark, the absorbance was recorded at 520 nm. The antioxidant activity was calculated using a Trolox calibration curve in linearity range of 10–200 μM (R^2^ = 0.99). The results are expressed as μmol Trolox equivalent (TE)/100 g sample.

### 2.8. Carotenoids Content

To 3 g of the samples was added 16 mL of n-hexane, and the mixture was vortexed for 1 min, sonicated for 30 min, and centrifugated at 3500 *g* for 10 min. The supernatant fractions were used for the determination of carotenoids using a Shimadzu UV 1601 spectrophotometer in a dark place (Milan, Italy) at a wavelength of 471 nm for total carotenoids (expressed as β-carotene eq.), 475 nm for β-carotene, and 502 nm for lycopene [23]. The absorbance value is derived from the respective concentration by applying the Lambert–Beer equation, using a molar extinction coefficient of 3150 dL g^−1^ cm for lycopene, 2049 dL g^−1^ cm for β-carotene, and 2049 dL g^−1^ cm for total carotenoids (expressed as β-carotene eq.) [25]. The results were calculated as mg/100 g of sample fresh weight (mg/100 g f.w.).

### 2.9. Total Dietary Fiber

Total dietary fiber (TDF) content was determined after enzymatic digestion combined with the gravimetric method as reported by Tagliamonte et al. [26].

### 2.10. Organic Acids and Ascorbic Acid Determination

The hydrophilic extracts (see Section 2.9) were also used for the organic acids (citric and malic acid) and ascorbic acid determination via an HPLC analysis. A chromatographic analysis was performed according to the methodology reported by Raiola et al. [18]. Briefly, the extracts were diluted to 30% *w/v* deionized water, filtered through a 0.45 µm filter, and injected in the HPLC system (Agilent 1100 series, Santa Clara, CA, USA) equipped with a degasser (G4225A), quaternary pump (G13111A), 20 µL loop, and diode array detector (G1315B). The column used was a Spherisorb ODS2 reverse phase (5 μm, 4.6 mm × 250 mm) (Waters, Arnhem, The Netherlands), while the eluents were water and phosphoric acid pH 2.0 (A) and methanol (B). The schedule involved a 3% isocratic injection of phase B at a constant flow rate of 0.8 mL for 20 min, and the identification of the sample and the standard was made at 210 nm. A calibration curve (10–300 ppm) was plotted for the organic acids and ascorbic acid. The limit of detection and limit of quantification were 1 and 5 ppm, respectively. The results were expressed as mg of individual compound/100 g of sample fresh weight (mg/100 g f.w.).

### 2.11. Color Measurement

The color of the tomato sauce was determined using a colorimeter (Chroma Meter, model CR-300; Minolta, Osaka, Japan). Colorimetric indices were measured, and the CIELab coordinates (L*, a*, b*) were recorded. The samples were poured into 90 cm diameter Petri dishes, and L* (lightness), a* (green-red direction), and b* (blue-yellow direction) color parameters were recorded.

### 2.12. Volatile Organic Compounds

Two grams of the sample were weighed into a 10 mL vial and kept at 60 °C for 10 min. To extract the volatile organic compounds (VOCs), a divinylbenzene/carboxene/polydimethylsiloxane (DVB/CAR/PDMS) fiber (Supelco, Bellefonte, PA, USA) was inserted into the vial and maintained at 60 °C for 10 min.

The vial was introduced into the gas chromatograph, and thermal desorption of the analytes was performed at 250 °C for 7 min in splitless mode. A gas chromatograph (GC) system equipped with a mass detector was purchased from Agilent (Santa Clara, CA, USA). VOC separation was performed according to the method described by Jiang et al. [27], with appropriate modifications. An HP-5MS 5% diphenyl/95% dimethylpolysiloxane capillary column (30 m × 0.25 mm ID × 0.25 µm) (Agilent) was used. The oven temperature was set at 40 °C for 2 min, then increased from 40 °C to 160 °C at 6 °C min^−1^ and from 160 °C to 210 °C at 10 °C min^−1^. The helium carrier gas was set at 1 mL/min. The ionizing electron energy was 70 eV, and mass-to-charge ratios were scanned in the range of 40–450 amu in full-scan acquisition mode. The injection and ion source temperatures were 250 and 230 °C, respectively. Compounds were identified by comparing mass spectra with those of the National Institute of Standards and Technology (NIST) atomic spectra database version 2.0 and verified for retention indices. The results are expressed as relative percentages (%) of VOCs.

### 2.13. Statistical Analysis

Each analysis was performed in triplicate, and the results are expressed as mean ± standard deviation. The data were submitted to analysis of variance (ANOVA) and Tukey’s test (*p* ≤ 0.05) using XLSTAT software version 2023 (Addinsoft, New York, NY, USA).

## 3. Results and Discussion

### 3.1. Evaluation of pH, °Brix, Viscosity, Ash, and Dry Matter

In Table 2, pH, °Brix, ash content, dry matter, and Bostwick viscosity values measured in the four types of tomato sauce were reported. All analyzed samples exhibited pH and °Brix values compliant with the G.U. 232/2005 [16], with pH ≤ 4.5 and °Brix ≤ 12.

Regarding the Bostwick, in the whole tomato sauces (WTS and WETS), a lower value in Bostiwick compared to traditional versions (TS and ETS) was found, indicating increased viscosity, probably due to the presence of peels and seeds.

The addition of vegetables did not significantly affect viscosity in both versions of the whole tomato sauce, while in the traditional sauce, the addition of vegetables increased the Bostiwich value with a reduction in viscosity.

The dry matter content was higher in whole tomato sauce (WTS and WETS) compared to traditional versions (TS and ETS), which also corresponded at samples with high °Brix and ash values. The high value of these parameters is attributable to the intrinsic composition of the peels and seeds because they are rich in insoluble fibers and polysaccharides, such as cellulose and hemicellulose, as well as minerals, which are relatively resistant to processing and tend to concentrate during evaporation [28]. Insoluble fibers contribute to total solids by remaining in the product after processing, while the minerals present naturally in peels and seeds elevate the ash content.

Additionally, soluble sugars and organic acids retained within the structural matrix of these fractions can increase the °Brix value, especially when milled during the process and thoroughly incorporated into the product [29,30].

### 3.2. Determination of Titratable Acidity, Reducing Sugars, and Fat Content

Table 3 shows the results of titratable acidity, reducing sugars, and fat content of the four samples. The enrichment with vegetables led to an increase in the sugar content for both the traditional (WTS) and the whole (WETS) versions due, therefore, to the addition of high-sugar vegetables, such as pumpkin and carrot, both added at a concentration of 5, which thus contribute a higher sugar content than tomatoes. As reported by Sharma & Ramana Rao [31] and Alasalvar et al. [32], the sugar contents of pumpkin and carrot are 10.65 and 5.47%, respectively, while, in the case of tomato, it is about 2–3% in Italian varieties as reported by [33]. The fat content increases in the WTS; this is due to the presence of seeds that contain about 20% of fat [34].

### 3.3. Mineral Determination

The mineral content of traditional tomato sauce (TS), traditional enriched tomato sauce (ETS), whole tomato Sauce (WTS), and whole enriched tomato sauce (WETS) was reported in Table 4.

The results showed that the whole versions of sauces (with peels and seeds of tomato) increased the content of minerals tested (calcium, sodium, magnesium, and potassium), as reported by Tagliamonte et al. [26], who show that switching from a traditional purée to a whole sauce results in a higher concentration of minerals. So, tomatoes appear to be a source of minerals as reported in several studies [35,36].

Anyway, the addition of vegetables to tomato sauce in both the traditional and whole variants reduced the mineral content, presumably due to the lower concentration of these elements in the vegetables used to enrich the recipe, and consequently, a decrease in the percentage of tomato also leads to a decrease in minerals [37].

The mineral salt content in tomato fruits, as well as in other vegetables, is known to be influenced by several factors, including crop variety, agricultural practices, and environmental conditions, particularly those related to the region of production [38].

It is important to emphasize that calcium and potassium, present in high concentrations in the whole sauce, perform important biological functions, including positively influencing the health of the immune system, blood pressure regulation, and muscle contraction.

Anyway, it is important to remember that only a certain amount of minerals are bioavailable. The bioavailability of macro- and micro-minerals is defined as the fraction of the ingested mineral that is absorbed and consequently used for usual physiological functions and is influenced by several factors, such as the composition of the food matrix and the formation of complexes with other compounds, such as polyphenols and fibers, and by the type of production process [39].

### 3.4. Total Phenolic Compounds and Antioxidant Activity

Table 5 shows the total polyphenol content (mgGAE/kg f.w.) of the four types of tomato sauce investigated and the antioxidant activity. The results show that the WETS sample had the highest TPC content, due to the combination of the addition of vegetables and tomato peels and seeds. Following this, the WTS sample showed a high concentration of TPC due to the presence of tomato peels and seeds, while ETS and TS showed lower values compared to whole versions. However, the ETS sample showed a statistically significant difference from TS due to the addition of vegetables, such as pumpkin and carrot, which provide antioxidant fiber and thus increase the content of bioactive compounds such as polyphenols. Polyphenols are phytocompounds [40] that show a strong antioxidant property and, in plant matrices, are anchored in the fiber [41]. During food processing, the interaction between polyphenols and polysaccharides is inevitable, as it influences the development of various microstructures by altering the food system’s physicochemical properties. These changes, in turn, impact the sensory qualities, nutritional value, and functional properties of the final product [42]. Marszałek et al. [43] evaluated the bioaccessibility of total polyphenols in apple juice subjected to high-pressure treatment, and the results showed that an increase in pressure leads to a greater release of polyphenols during in vitro digestion, as the bioaccessibility of polyphenols in the control sample (not subjected to high pressure) was 36.8%, while the samples subjected to high pressure reached 51.3–55.8%. This result may be due to damage to the cell paratheses that cause greater release of polyphenols. Regarding the antioxidant activity, the results show how the WETS sample showed the highest antioxidant activity values for both assays. In general, the addition of vegetables present in the recipe leads to an increase in antioxidant activity. In fact, both the ETS and WETS samples showed higher values than the TS and WTS variants, respectively. The ETS sample showed an increase of about 15% compared to TS, with values rising from 76.71 to 66.69 (µmol TE/100 g), 381.34 to 328.96 (µmol TE/100 g), and 165.10 to 150.30 (mg GAE/kg) in the DPPH, ABTS, and FRAP assays, respectively. Similarly, the WETS sample showed an average increase of about 8% relative to the WTS sample, with corresponding increases from 87.07 to 80.61 (µmol TE/100 g), 395.61 to 362.70 (µmol TE/100 g), and 270.40 to 250.22 (mg GAE/kg) for the DPPH, ABTS, and FRAP assays, respectively. These results are in agreement with the TPC content, whereby the WETS sample showed the highest polyphenol content and consequently also exhibited the highest antioxidant activity.

### 3.5. Total Carotenoids

**Figure 2:**
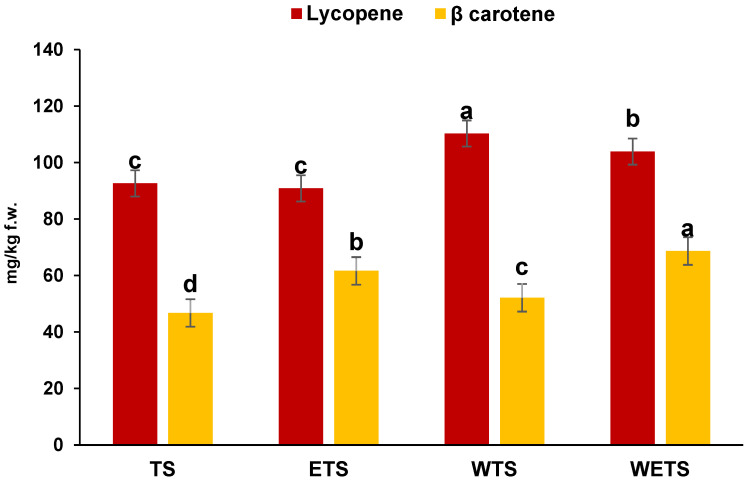
Lycopene and β-carotene content (mg/kg f.w.) of traditional tomato sauce (TS), Traditional Enriched tomato sauce (ETS), Whole tomato sauce (WTS) and Whole Enriched tomato sauce (WETS). ^a–d^ Different letters in the same column indicate significant difference (*p* < 0.05). Data represent the mean ± standard deviation of three replications (*n* = 3).

Figure 2 shows the content of lycopene and β-carotene in tomato sauce. The results showed that the integration of tomato peels in whole tomato sauce and whole tomato (WTS) and whole enriched tomato sauce (WETS) has led to a significant increase in lycopene content. Specifically, in traditional sauce (TS), an increase of 22% for lycopene was observed, compared to whole sauce (WTS). As previously reported, tomato peels are among the main sources of lycopene, and their incorporation into the sauce directly contributes to the enhancement of this compound. In line with previous findings, mechanical homogenization treatments can improve the release and bioavailability of carotenoids [37]. This phenomenon is primarily due to the breakdown of the cell structure in peels, which facilitates the release of lycopene from the plant matrix, thereby increasing its absorption [44]. Similarly, a comparable trend was observed in the WETS, where the combination of tomato peels and vegetables maintained a high level of lycopene compared to the traditional sauce. Regarding the β-carotene content, the results revealed significant differences in content among the various analyzed samples. In increasing order, the lowest concentration was found in TS followed by WTS, ETS, and WETS. These findings indicate that enriched formulations contain significantly higher β-carotene levels (*p* < 0.05) compared to the unenriched version. This difference can be primarily attributed to the presence of carrots and pumpkin, which are well-known sources of β-carotene [45]. Specifically, the β-carotene content in ETS was 61.66 mg/kg of f.w., a higher value compared to TS, which contained 46.76 mg/kg of f.w. Similarly, the vegetable-enriched whole sauce showed a β-carotene concentration of 68.67 mg/kg of f.w., exceeding that of the whole sauce, which had a content of 52.13 mg/kg of f.w. Although enrichment enhances the potential functional properties of tomato sauce compared to the traditional, it also leads to a slight reduction in total carotenoid content. This decrease is a direct consequence of the addition of vegetables, which results in a reduction percentage of tomatoes in the formulation. These findings align with [46], who observed that in tomato and pumpkin sauce, the total carotenoid content is primarily influenced by the proportion of tomato used in the formulation.

### 3.6. Total Dietary Fiber

In Table 6, the content of total dietary fiber (TDF) was reported. The data showed the highest value in whole tomato sauce in both unenriched (WTS) and enriched versions (WETS). This could be due to the presence of the peels in the case of the unenriched version and also the vegetables in the case of the enriched version. In this way, [47] reported that the content of TDF in tomato peels dried was 75%. In addition, the dietary fiber content is about 58g/100 g d.w and 23g/100 g d.w in pumpkin and carrot, respectively [48,49]. The WTS and WETS samples show a fiber content greater than 3g/100 g, and consequently, the “Source of Fiber” claim can be included [50]. TDF refers to the component of plant material that is resistant to digestion in the human large intestine [51]. Extensive research has established numerous beneficial effects of TDF on human health and physiological functioning [8,52,53]. Therefore, a higher intake of TDF is associated with a decreased incidence of prevalent disorders and diseases in developed nations, including chronic bowel disorders, obesity, diabetes, cardiovascular diseases, and cancer [8].

### 3.7. Organic Acid and Ascorbic Acid Determination

In Table 7, the content of organic acid (malic and citric acid) and vitamin C was reported. The major organic acids in tomato are malic acid and citric acid [54]. For malic acid, the ANOVA showed no statistically significant differences between the samples (*p* > 0.05), as indicated by the statistical analysis in Table 7. Therefore, neither enrichment nor the addition of skins and seeds seems to influence the malic acid content. The citric acid concentration was highest in traditional tomato sauce (TS) followed by whole tomato sauce (WTS), while in the enriched sauces, the content is lower. This result could be due to the substitution of tomatoes, in which citric acid is the predominant organic acid, by vegetables such as pumpkin and carrots, which have a lower content of this acid. Indeed, the concentration of citric acid in pumpkin is a highly variable and dependent on storage time and the cultivar analyzed [55]. The citric acid content of carrots also varies depending on the cultivar analyzed in a range from 0 to 140 mg/100 g as reported by Yusuf et al. [56]. The ascorbic acid content ranges between 7.05 and 11.36 mg/100 g f.w. This result was lower than Chanforan et al.’s results [57], which reported an ascorbic acid content of 27 mg/100 g f.w. in tomato paste, while [58] reported a content of about 5 mg/100 g f.w. in tomato juice.

The different concentrations, the applied technologies, and the raw materials were different; in particular, a large difference is reported depending on the variety and ripening stage of the tomato [59], the cultivation method, the sampling period [60], and the type of production process [61]. The WTS sample showed the highest ascorbic acid content, followed by WETS, TS, and ETS. The addition of peels and seeds lead to an increase in Ascorbic Acid, due to the higher concentration within the peel [52]. In addition, enrichment led to a decrease in tomato sauce, with enrichment showing no statistically significant differences with WTS, confirming the greater contribution made by peels compared to vegetables in terms of ascorbic acid content [49], who show that in the formulation of various tomato and pumpkin sauces, increasing the amount of tomatoes revealed a higher concentration of ascorbic acid. L-ascorbic acid is one of the most reactive compounds present and is therefore particularly susceptible to processing and storage conditions [62,63].

### 3.8. Color Evaluation

The color indices are reported in Table 8. The L* color coordinate represents lightness, with values ranging from 0 (black) to 100 (white). The a* coordinate indicates chromaticity along the red–green axis, where positive values correspond to red and negative values to green. Similarly, the b* coordinate reflects chromaticity along the yellow–blue axis, with yellow indicating positive values and blue indicating negative values [64]. The value of a* ranged between 20.01 and 24.36, whereas the value of b*, an indicator of the yellow color, ranged from 34.88 to 56.35. Both parameters a* and b* were found to be significantly high in the WTS and WETS sample, due to the addition of tomato peels and seeds, which consequently increase parameter a* for the addition of lycopene-rich peels and parameter b* for the addition of seeds. The ETS sample showed the lowest a* values of all the samples, which is due to the addition of the other vegetables such as pumpkin and carrot, which are higher in β-carotene than lycopene. A similar result was also shown by Rahman et al. [46], where they formulated various tomato and pumpkin-based sauces and demonstrated that increasing the amount of tomato leads to an increase in the a* parameter. The authors found an increase in the a* parameter of about 74% in the sauce made from 100% tomato compared to the sauce made from 100% pumpkin.

García et al. [65] evaluated the addition of dried tomato skins inside hamburgers and assessed the change in color as a function of tomato skin concentration. As the content increased, the a* parameter also increased, confirming that the lycopene content inside peels also increases the red coloration of the product.

### 3.9. Volatile Organic Compounds

In Table 9, the VOCs content in samples were reported. The most abundant chemical class is ketones, followed by terpenes, aldehydes, and alcohols for a total of 24 compounds. Among the ketones, the most abundant compound is 6-methyl-5-heptene-2-one, in agreement with the data reported by Romano et al. [4]. In the whole enriched tomato sauce (WETS), the highest concentration was found followed by whole tomato sauce (WTS), traditional enriched tomato sauce (ETS), and traditional tomato sauce (TS). This molecule represents one of the major molecules in tomato products [66], responsible for herbaceous, floral, and pungent notes [67,68], and it is derived from lycopene metabolism [69]. In fact, it is precisely the whole sauce versions in which a higher concentration of this compound has been shown, as they contain the peels known to have a high lycopene content. Hexanal was found to be one of the most prevalent aldehydes in all four samples with a higher concentration in WTS and TS, and this compound appears to be one of the most prevalent VOCs present in fresh tomatoes [59,70]. Furfural is generally not found in fresh tomatoes but rather within processed tomato products, and consequently, its presence is related to heat treatment [71]; it was found at high concentrations in the whole sauce versions, and this is probably due to the presence of seeds. In fact, it has been reported that this compound is present in tomato seed oil [72]. The formulation of the ETS and WETS that then included the addition of other botanicals resulted in an increase in terpene content (eucalyptol, terpinen-4-ol, thymol, and borneol are the four molecules found only in vegetable-enriched versions of tomato sauce) compared with the respective unenriched variant. The results show that there was a 4.35% increase in the total terpene content for the ETS sample compared with TS, while for the WETS sample, there was a 10.27% increase compared with WTS. The presence of compounds such as eucalyptol, 4-terpineol, borneol L, α-terpineol, and thymol may be associated with the presence of rosemary [73] and basil [74].

Among alcohols, the most present were 3-hexen-1-ol and 1-pentanol, which influence the odor, green color, and sweetness of tomato products [4,66,75]. Anyway, 1-pentanol at high concentration could impart an undesirable flavor [76]. Furthermore, the enrichment with other vegetables reduced the concentration of these compounds, reducing the risk of undesirable aroma formation.

So, the enrichment with other vegetables greatly influenced the concentration of terpenes and ketones in the tomato sauce, leading to greater aromatic complexity, presumably influencing the product aroma by imparting minty, flowery, and spicy notes [77,78]. However, these data will have to be confirmed by more specific sensory analyses.

## 4. Conclusions

This study demonstrates how incorporating tomato peels, seeds, and vegetables can significantly boost the nutritional value of tomato sauce, particularly by increasing lycopene and β-carotene levels. These results suggest that enriched tomato products could meet the growing demand for healthier, more functional foods among consumers. From an industry perspective, repurposing tomato by-products offers a smart and cost-effective way to enhance nutritional quality without increasing production costs. Additionally, modern processing techniques like homogenization could further improve carotenoid absorption, making these enriched formulations more appealing for large-scale production. On the sustainability front, using tomato peels and seeds aligns with the principles of circular economy, helping to reduce food waste and minimize the environmental footprint of tomato processing. This approach supports a more eco-friendly and resource-efficient food industry, making better use of existing raw materials. However, further studies are needed to evaluate the sensory impact of such enrichments, as consumer acceptance plays a crucial role in product success. In this regard, incorporating a sensory analysis in future research would provide valuable insights into the overall appeal of the enriched sauces. Moreover, exploring the scalability, shelf life, and potential market applications of these enriched products could support their transition from research to commercial development. However, a limitation of this study may be the absence of a sensory analysis to assess the overall acceptability of the product. By addressing these aspects, this work could open new doors for functional food innovations, making nutrient-rich, sustainable tomato products more accessible to consumers worldwide.

## Figures and Tables

**Table 1:** Percentage of ingredients in the enriched tomato sauces: ETS (traditional enriched sauce) and WETS (whole enriched tomato sauce).

Ingredient (%)	ETS/WETS
Tomato	85.7
Pumpkin	5.0
Carrot	5.0
Onion	3.0
Oregano	0.1
Basil	1.2

**Table 2:** Values of pH, °Brix, Bostwick, dry matter (g/100 g), and ash (g/100 g) of the traditional tomato sauce (TS), traditional enriched tomato sauce (ETS), whole tomato sauce (WTS), and whole enriched tomato sauce (WETS).

Sample	pH	°Brix	Bostwick	Dry Matter %	Ash %
TS	4.21 ± 0.01 ^c^	7.75 ± 0.07 ^b^	6.01 ± 0.01 ^b^	8.35 ± 0.07 ^c^	0.84 ±0.04 ^bc^
ETS	4.29 ± 0.01 ^a^	8.15 ± 0.07 ^a^	6.15 ± 0.07 ^a^	8.38 ± 0.07 ^c^	0.76 ± 0.02 ^c^
WTS	4.24 ± 0.02 ^b^	7.70 ± 0.01 ^b^	5.50 ± 0.07 ^d^	8.89 ± 0.01 ^a^	0.95 ± 0.05 ^a^
WETS	4.20 ± 0.01 ^c^	8.05 ± 0.07 ^a^	5.70 ± 0.14 ^cd^	8.61 ± 0.02 ^b^	0.87 ±0.01 ^ab^

^a–d^ Different letters in the same column indicate significant difference (*p* ≤ 0.05). The data represent the mean ± standard deviation of three replications (*n* = 3).

**Table 3:** Titratable acidity (%), reducing sugars (%), and fat content (%) of traditional tomato sauce (TS), traditional Enriched tomato sauce (ETS), whole tomato sauce (WTS), and whole enriched tomato sauce (WETS).

Sample	Titratable Acidity (%)	Reducing Sugars (%)	Fat Content (%)
TS	0.56 ± 0.01 ^a^	2.69 ± 0.02 ^d^	0.16 ± 0.01 ^b^
ETS	0.56 ± 0.01 ^a^	4.04 ± 0.15 ^a^	0.11 ± 0.03 ^b^
WTS	0.46 ± 0.01 ^b^	2.96 ± 0.01 ^c^	0.25 ± 0.01 ^a^
WETS	0.42 ± 0.00 ^b^	3.33 ± 0.05 ^b^	0.13 ± 0.03 ^b^

^a–d^ Different letters in the same column indicate significant difference (*p* ≤ 0.05). The data represent the mean ± standard deviation of three replications (*n* = 3).

**Table 4:** Mineral content (mg/100 g f.w.) of traditional tomato sauce (TS), traditional enriched tomato sauce (ETS), whole tomato sauce (WTS), and whole enriched tomato sauce (WETS).

Sample	Ca	Na	Mg	K
TS	29.35 ± 1.58 ^c^	75.36 ± 1.68 ^b^	16.88 ± 0.300 ^a^	322.1 ± 1.78 ^b^
ETS	27.96 ± 0.30 ^c^	71.22 ± 0.65 ^c^	14.40 ± 0.384 ^b^	284.1 ± 0.97 ^c^
WTS	43.21 ± 1.23 ^a^	79.73 ± 1.86 ^a^	17.58 ± 0.517 ^a^	381.2 ± 1.23 ^a^
WETS	37.93 ± 0.96 ^b^	77.71 ± 0.64 ^ab^	15.30 ± 0.081 ^b^	305.5 ± 0.51 ^bc^

^a–c^ Different letters in the same column indicate significant difference (*p* ≤ 0.05). The data represent the mean ± standard deviation of three replications (*n* = 3).

**Table 5:** Antioxidant activity determined by spectrophotometric DPPH and ABTS assays of traditional tomato sauce (TS), traditional enriched tomato sauce (ETS), whole tomato sauce (WTS), and whole enriched tomato sauce (WETS).

Sample	DPPH (µmol TE/100 g)	ABTS (µmol TE/100 g)	TPC (mg GAE/kg f.w.)
TS	66.96 ± 1.31 ^d^	328.96 ± 0.22 ^d^	150.30 ± 2.81 ^d^
ETS	76.71 ± 0.97 ^c^	381.34 ± 0.37 ^b^	165.10 ± 1.26 ^c^
WTS	80.61 ± 1.85 ^b^	362.70± 0.12 ^c^	250.22 ± 2.33 ^b^
WETS	87.07 ± 0.40 ^a^	395.61 ± 0.81 ^a^	270.40 ± 2.68 ^a^

^a–d^ Different letters in the same column indicate significant difference (*p* ≤ 0.05). The data represent the mean ± standard deviation of three replications (*n* = 3).

**Table 6:** Total fiber content (%) of traditional tomato sauce (TS), traditional enriched tomato sauce (ETS), whole tomato sauce (WTS), and whole enriched tomato sauce (WETS).

Sample	TDF %
TS	2.93 ± 0.32 ^b^
ETS	2.78 ± 0.17 ^b^
WTS	4.55 ± 0.94 ^a^
WETS	3.95 ± 0.10 ^ab^

^a, b^ Different letters in the same column indicate significant difference (*p* < 0.05). The data represent mean ± standard deviation of three replications (*n* = 3).

**Table 7:** Organic acid and vitamin C content (mg/100 g f.w.) of traditional tomato sauce (TS), traditional enriched tomato sauce (ETS), whole tomato sauce (WTS), and whole enriched tomato sauce (WETS).

Sample	Ascorbic Acid	Malic Acid	Citric Acid
TS	7.70± 0.23 ^b^	179.69 ± 1.41 ^a^	301.73 ± 0.61 ^a^
ETS	7.05 ± 0.19 ^c^	175.45 ± 3.14 ^a^	294.92 ±2.45 ^b^
WTS	11.36 ± 0.17 ^a^	177.22± 1.56 ^a^	298.39 ± 1.80 ^ab^
WETS	11.01 ± 0.22 ^a^	173.86± 0.92 ^a^	293.9 ± 1.07 ^b^

^a–c^ Different letters in the same column indicate significant difference (*p* < 0.05). The data represent mean ± standard deviation of three replications (*n* = 3).

**Table 8:** Colorimetric indices (L*, a*, b*) of traditional tomato sauce (TS), traditional enriched tomato sauce (ETS), whole tomato sauce (WTS), and whole enriched tomato sauce (WETS).

Sample	L*	a*	b*
TS	37.27 ± 0.37 ^a^	23.16 ± 0.93 ^b^	34.88 ± 1.45 ^b^
ETS	40.84 ± 0.61 ^a^	20.01 ± 0.30 ^c^	35.06 ± 0.16 ^b^
WTS	28.87 ± 0.62 ^b^	24.36 ± 0.30 ^a^	56.35 ± 0.00 ^a^
WETS	26.11 ± 0.25 ^b^	24.19 ± 0.13 ^a^	54.87 ± 0.05 ^a^

^a–c^ Different letters in the same column indicate significant difference (*p* ≤ 0.05). The data represent the mean ± standard deviation of three replications (*n* = 3).

**Table 9:** Volatile organic compound (%) of traditional tomato sauce (TS), traditional enriched tomato sauce (ETS), whole tomato sauce (WTS), and whole enriched tomato sauce (WETS).

	TS	ETS	WTS	WETS
**ΣAlcohols**	**5.16 ± 0.54 ^b^**	**2.26 ± 0.98 ^d^**	**15.53 ± 1.54 ^a^**	**3.44 ± 0.83 ^c^**
1-Pentanol	0.43 ± 0.04 ^a^	n.d.	0.48 ± 0.05 ^a^	0.27 ± 0.03 ^b^
(*Z*)-3-Hexen-1-ol	4.19 ± 0.63 ^b^	2.21 ± 0.42 ^c^	12.87 ± 1.35 ^a^	2.14 ± 0.36 ^c^
3-Cyclohexen-1-ol, 4-methyl	0.54 ± 0.06 ^b^	0.07 ± 0.01 ^c^	0.88 ± 0.01 ^a^	1.03 ± 0.22 ^a^
1-octanol	n.d.	n.d.	1.30 ± 0.26 ^a^	n.d.
**ΣTerpens**	**24.77 ± 1.13 ^b^**	**28.49 ± 1.89 ^a^**	**9.32 ± 0.94 ^d^**	**20.17 ± 1.86 ^c^**
α-terpineol	11.61 ± 1.42 ^a^	7.64 ± 0.92 ^b^	n.d.	n.d.
Linalool	3.53 ± 0.52 ^c^	5.45 ± 0.51 ^b^	7.49 ± 0.42 ^a^	7.64 ± 0.56 ^a^
β-Damascenone	7.99 ± 0.62 ^a^	n.d.	1.13 ± 0.42 ^b^	n.d.
Methyleugenol	n.d.	2.36 ± 0.27 ^a^	n.d.	n.d.
Eucalyptol	n.d.	0.61 ± 0.08 ^b^	n.d.	1.22 ± 0.37 ^a^
(-)Terpinen-4-ol	1.09 ± 0.23 ^a^	3.11 ± 0.36 ^b^	0.54 ± 0.05 ^b^	4.09 ± 0.32 ^a^
Thymol	n.d.	3.83 ± 0.45 ^a^	n.d.	1.59 ± 0.32 ^b^
Borneol	n.d.	5.10 ± 0.82 ^a^	n.d.	3.45 ± 0.42 ^a^
Eugenol	0.55 ± 0.08 ^bc^	0.39 ± 0.04 ^c^	0.16 ± 0.04 ^d^	2.18 ± 0.47 ^a^
**ΣAldehydes**	**20.54 ± 1.13 ^a^**	**13.37 ± 1.01 ^b^**	**20.97 ± 1.28 ^a^**	**15.27 ± 1.14 ^b^**
2-(Hexyloxy)benzaldehyde	5.78 ± 0.81 ^a^	4.14 ± 0.77 ^a^	2.34 ± 0.56 ^b^	2.15 ± 0.39 ^b^
Hexanal	7.78 ± 0.78 ^a^	4.90 ± 0.71 ^b^	8.42 ± 0.66 ^a^	6.14 ± 0.82 ^ab^
Benzeneacetaldehyde	2.24 ± 0.31 ^a^	1.84 ± 0.22 ^a^	1.64 ± 0.26 ^ab^	0.89 ± 0.10 ^b^
Furfural	4.72 ± 0.63 ^b^	2.48 ± 0.33 ^c^	8.56 ± 0.88 ^a^	6.07 ± 0.91 ^a^
**ΣKetones**	**46.27 ± 1.33 ^c^**	**54.01 ± 2.26 ^ab^**	**54.18 ± 2.88 ^b^**	**61.12 ± 3.50 ^a^**
6-methyl-5-heptene-2one	43.18 ± 1.52 ^c^	48.81 ± 1.31 ^b^	51.13 ± 1.55 ^b^	56.31 ± 1.59 ^a^
Geranylacetone	3.09 ± 0.81 ^b^	5.20 ± 0.91 ^a^	3.04 ± 0.78 ^b^	4.34 ± 0.66 ^ab^
(+)2-Bornanone	n.d.	n.d.	n.d.	0.47 ± 0.03
**ΣOther**	**3.26 ± 0.51 ^a^**	**1.87 ± 0.48 ^b^**	**n.d.**	**n.d.**
1,3-Di-terz-butylbenzene	0.52 ± 0.09 ^a^	n.d.	n.d.	n.d.
2,3-Di-tert-butylphenol	2.69 ± 0.71 ^a^	0.73 ± 0.10 ^b^	n.d.	n.d.
5-Methylindole	n.d.	1.14 ± 0.33 ^a^	n.d.	n.d.

^a–d^ Different letters in the same rows indicate significant difference (*p* < 0.05). The data represent mean ± standard deviation of three replications (*n* = 3); n.d.: not detected.

## Data Availability

Data is contained within the article.
